# Uniaxial Compressive Constitutive Relationship of Concrete Confined by Special-Shaped Steel Tube Coupled with Multiple Cavities

**DOI:** 10.3390/ma9020086

**Published:** 2016-01-29

**Authors:** Haipeng Wu, Wanlin Cao, Qiyun Qiao, Hongying Dong

**Affiliations:** College of Architecture and Civil Engineering, Beijing University of Technology, Beijing 100124, China; caowl@bjut.edu.cn (W.C.); qiaoqiyun@bjut.edu.cn (Q.Q.); donghy@bjut.edu.cn (H.D.)

**Keywords:** constitutive relationship, confined concrete, special-shaped cross-section, concrete filled steel tube (CFT), multiple cavities

## Abstract

A method is presented to predict the complete stress-strain curves of concrete subjected to triaxial stresses, which were caused by axial load and lateral force. The stress can be induced due to the confinement action inside a special-shaped steel tube having multiple cavities. The existing reinforced confined concrete formulas have been improved to determine the confinement action. The influence of cross-sectional shape, of cavity construction, of stiffening ribs and of reinforcement in cavities has been considered in the model. The parameters of the model are determined on the basis of experimental results of an axial compression test for two different kinds of special-shaped concrete filled steel tube (CFT) columns with multiple cavities. The complete load-strain curves of the special-shaped CFT columns are estimated. The predicted concrete strength and the post-peak behavior are found to show good agreement within the accepted limits, compared with the experimental results. In addition, the parameters of proposed model are taken from two kinds of totally different CFT columns, so that it can be concluded that this model is also applicable to concrete confined by other special-shaped steel tubes.

## 1. Introduction

Concrete filled steel tubes (CFTs) combine steel and concrete, which results in tubes that have the beneficial qualities of high tensile strength and the ductility of steel as well as the high compressive strength and stiffness of concrete. Hence, they possess perfect seismic resistance property. In recent years, mega-frame structures have widely been applied to super high-rise buildings for their clear force transferring paths between primary and secondary structures, and flexible arrangement of structural members [1,2]. To fulfill the requirements of structural safety, architectural layout and economic efficiency, the mega CFT (concrete filled steel tube) columns are often designed as special shapes with multiple cavities, which are often very different from normal circular and rectangular CFTs [3,4,5].

The constitutive relationships play a pivotal role for both design and research in concrete materials and structures. Due to the diversity of concrete materials, inconformity of test methods and confinement of steel tubes, various stress-strain equations have been proposed in the past; most of them originated from classical theories. These models can be divided into two types, *i.e.*, the uniaxial and the triaxial. The constitutive relationships for the case of uniaxial model are simple and are often used in fiber based models; whereas, for the case of triaxial models, these relationships are complex and are often used in finite element methods (FEMs) [6,7]. A typical stress-strain curve of steel tube confined concrete exhibits an ascending trend followed by a post-peak descending behavior [8,9,10]. In the case of circular tube confined concrete, Tang *et al.* [11] have established a stress-stain relationship for circular steel tube confined concrete by assuming the steel ratio, the tube width-to-thickness ratio and the material’s property on the strength and post-peak behavior. Xiao [12] conducted a series of tests on concrete filled steel tube stub columns, and then proposed a triaxial constitutive relationship, in which failure criterion and flow rule are expressed by octahedron element. Zhong and Han *et al.* [13,14] have conducted a number of tests on circular and rectangular CFT columns and established the constitutive relationships, which were derived by using regression analysis. Chen [15] has developed and modified the increment constitutive relationships based on plastic-fracturing mechanics, which were initially proposed by Banzant [16]. Susantha *et al.* [17] have proposed the calculation method of stress-strain relationships of CFT-subjected axial load and horizontal load. In this model, the existing formulas and the FEM method have been used to determine the pressure of confinement for steel tube to core concrete. In the cases of square and rectangular steel tube confined concrete, Hajjar *et al.* [18] have developed a triaxial constitutive relationship that was expressed in a polynomial order; this model can be used to estimate the behavior of CFT under the coupled effect of axial force and bending moment. Watanabe *et al.* [19] have proposed a stress-strain relationship, which is applicable to a rectangular CFT; in this model, the local buckling of component plate and initial imperfection are considered. Tomii *et al.* [20] have proposed a stress-strain relationship of concrete confined by square steel tubes; the ascent stage of the model adopted second-degree parabola, whereas the cylindrical strength is assumed as peak strength. On the basis of Mander [21], Long and Cai *et al.* [22] have established new models for confined concrete especially for the concrete confined by rectangular steel tubes along with binding bars.

The above mentioned literature is only limited to either circular or rectangular steel tube confined concrete. Only few cases have been reported regarding the constitutive relationships of concrete that was confined by special-shaped steel tube with multiple cavities. The confinement action of special-shaped steel tubes with multiple cavities is different than that of normal steel tubes (either circular or rectangular including the quadratic shape). Hence, the constitutive relationships of concrete confined by normal steel tubes, available in the literature, are not suitable for the concrete confined by special-shaped steel tube with multiple cavities. The confinement pressure, to the core concrete in special-shaped steel tube with multiple cavities, can be determined by evaluating the cross-sectional shape, cavity construction, steel ratio of outer steel tube and inner cavity partition steel plates, steel ribs, steel bars in cavities, *etc.* It is very complex to estimate what extent confinement action can be considered. On the basis of an axial compression test of two groups of special-shaped CFT columns with multiple cavities, this article evaluates how each factor contributes to confinement action of core concrete, and proposes uniaxial stress-strain relationship based on Mander’s model. The theoretical results match well with the test results.

## 2. Model of Constitutive Relationship 

### 2.1. Confinement Mechanism

The normal CFT and special-shaped CFT coupled with multiple cavities may lead to generate longitudinal deformation as well as transversal deformation under axial loading. Owing to the fact that the Poisson’s ratio of concrete is smaller than that of steel during the initial loading stage, the steel tube and in-filled concrete make a trend of departure and there’s no squeezing between them. When the stress of steel tube is loaded to reach its proportional limit, the Poisson’s ratio of concrete is approximately equal to that of steel. When the stress of steel tube exceeds to its proportional limit, the Poisson’s ratio of concrete is greater than that of steel; a lateral interactional force generates along with squeezing trend between them, owing to the fact that the steel tube constrains concrete transversal deformation.

The confinement action in case of a circular CFT is, generally, better than that of a rectangular CFT; whereas, it is of medium order for the case of regular polygonal CFT. It is strongly increased as the side number of a CFT increases. It also differs when the cross-section changes from a regular shape to an irregular shape. The research [14] shows that the confinement action at the corner of steel tube is strong, whereas it is weak at the central part of the sides of steel tube. It is pertinent to note that the confinement action increases in the case of a small interior angle that may be formed by the adjacent steel plates. In the case of a special-shaped CFT coupled with multiple cavities, the inner partition steel plate can effectively be used to reduce the interior angle, as well as to provide transverse constraint for the external steel tube. As a consequence, the confinement action of a special-shaped CFT coupled with multiple cavities is strengthened, and the bearing capacity and ductility are improved.

**Figure 1:**
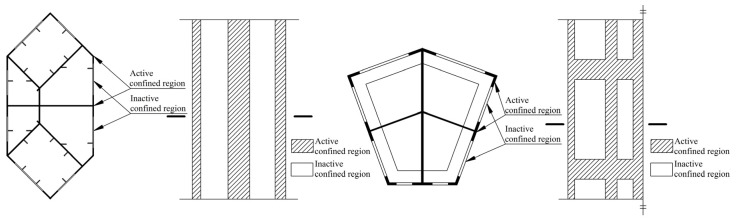
Division of confined regions of special-shaped concrete filled steel tube (CFT) column with multiple cavities.

Analogous with normal reinforced confined concrete, the external steel tube of a special-shaped CFT coupled with multiple cavities performs as longitudinal reinforcement and transverse reinforcement, while the inner partition steel plate performs as longitudinal reinforcement and tie bar. As it has been shown in Figure 1, the steel plate in longitudinal active confined region is equivalent to longitudinal reinforcement in normal confined concrete, whereas the steel plate in transverse active confined region is equivalent to tightened transversal reinforcement. Of course, the inactive confined region has a similar confinement effect which is relatively weak. In this article, the equivalent method of lateral confining stress proposed by Mander has been applied to study the confinement action for a special-shaped CFT coupled with multiple cavities. The difference from Mander’s confined concrete model is that the special-shaped steel tube with multiple cavities does not bear only longitudinal force but also transversal force. It is a three dimensional complex stress status, and it is compressed in longitudinal and radial directions, whereas it is pulled in a hooping direction. Keeping this in view, the influence of longitudinal stress to hooping stress needs to be considered in the theoretical estimation of an equivalent lateral confining stress. Zhong and Shamugam [13,23] have found regarding the circular CFT that the radial stress of steel tube is relatively small as compared to longitudinal and hooping stresses, and it can be neglected. Next, the confining stress of a special-shaped CFT coupled with multiple cavities is complex and variable; its values are different at different locations, e.g., at the corner, and at the center at the stiffening ribs of different sides. In this article, an equivalent average lateral confining stress is applied to simplify the situation and to reflect the confinement action of a special-shaped CFT coupled with multiple cavities.

### 2.2. The Proposed Model

An expression (unified) of an equivalent uniaxial stress-strain relationship of a concrete, which is confined by special-shaped CFT coupled with multiple cavities has been proposed. It is mainly based on Mander’s confined concrete model. The five parameter based strength criterion, as determined by William-Warnke, is applied to evaluate the ultimate strength of a confined concrete. The Popovics concrete stress-strain curve has been applied to express the constitutive relationship. The model can well reflect the characteristic of confined concrete that the strength and the strain at maximum concrete stress increase, while the descending branch tends to slow. The expressions can be described as follows:

(1) $$f_{c}=\frac{f_{\text{cc}}xr}{r-1+x^{r}}$$

(2) $$x=\frac{\varepsilon_{c}}{\varepsilon_{\text{cc}}}$$

(3) $$\varepsilon_{\text{cc}}=\varepsilon_{\text{c}0}[1+\eta (\frac{f_{\text{cc}}}{f_{\text{c}0}}-1)]$$

(4) $$r=\frac{E_{c}}{E_{c}-f_{\text{cc}}/\varepsilon_{\text{cc}}}$$

(5) $$f_{\text{cc}}=f_{\text{c}0}(-1.254+2.254\sqrt{1+\frac{7.94f_{l}}{f_{\text{c}0}}}-2\frac{f_{l}}{f_{\text{c}0}})$$

where, *f*_c_ and ε_c_ are the longitudinal compressive stress and strain of a core concrete respectively; *f*_c0_, $\varepsilon_{\text{co}}=\left(700+172\sqrt{f_{\text{co}}}\right)\times 10^{-6}$ and $E_{c}=\frac{10^{5}}{2.2+34.7/f_{\text{cu,k}}}$ [24] are the longitudinal compressive strength, the corresponding strain and the elasticity modulus of an unconfined concrete respectively; *f*_cc_ and ε_cc_ are the longitudinal compressive strength and corresponding strain of a concrete that is confined by special-shaped steel tube with multiple cavities, respectively; η is the correction coefficient of the strain at maximum concrete stress; γ is the shape parameter of the curve; *f*_l_ is an equivalent lateral confining stress.

Mander *et al.* have suggested “η = 5” for a concrete confined by reinforcement [22]. However, the correction coefficient is not constant for the concrete that is confined by a special-shaped CFT with multi-cavities in accordance with experimental research.

## 3. Results and Discussion

### 3.1. Experimental Test Data

The special-shaped CFTs coupled with multiple cavities are often applied in specific super high-rise buildings; the related experimental test data are seldom available. Only data of tests conducted by the authors of this paper are documented and available for review. Therefore, this article only focuses to discuss the data of axial compressive test for the six special-shaped CFT columns with multiple cavities, as these were conducted by the authors, to study an equivalent uniaxial stress-strain relationship for a confined concrete. By considering the cross-sectional shape, the cavity construction, the concrete strength, the steel strength, and the reinforcement arrangement in cavities differ in each column, the equivalent uniaxial stress-strain relationship that worked out from the test results has good applicability.

#### 3.1.1. Construction Details

Six special-shaped CFT columns coupled with multiple cavities were designed in accordance with actual CFT columns in super high-rise buildings. The six columns were divided into two groups as follows: (1) the group P that includes three irregular pentagonal CFT columns coupled with multiple cavities, and (2) the group H that includes three irregular hexagonal CFT columns coupled with multiple cavities. The scales of group P columns and group H columns, respectively, are 1/5 and 1/12. The real prototype mega column of group P has a cross sectional area of 45 m^2^, whereas group H is approximately equal to 9 m^2^. All the specimens were designed by using the geometric similarity principle.

**Figure 2:**
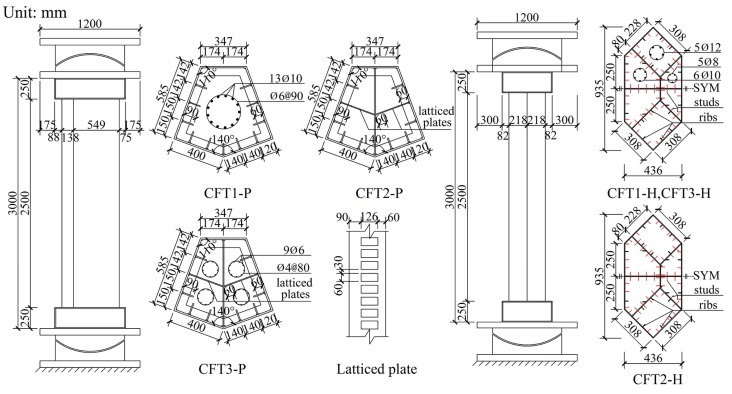
Column construction details.

**Figure 3:**
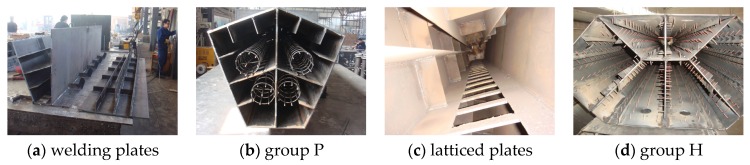
Column construction pictures.

Group P columns were named CFT1-P, CFT2-P and CFT3-P, respectively. The cross-sectional geometric dimensions of external steel tubes that were welded by 12 mm steel plates are same. The vertical continuous stiffening ribs, whose cross sectional dimensions were 90 mm × 6 mm, were welded to an inside face of an external steel tube, whereas five story horizontal continuous stiffening ribs were welded at a vertical spacing of 500 mm. For the case of the columns CFT2-P and CFT3-P, the cross-section was divided into two cavities by using a 10 mm thick solid partition steel plate, and, thereafter, it was divided into four cavities by using two 6 mm thick symmetrical lattice partition steel plate on which rectangular holes were punched to make it easy for flow of concrete between the adjacent cavities. Group H specimens were named CFT1-H, CFT2-H, CFT3-H, respectively. The cross-sectional geometric dimensions of steel tubes, which were welded into a hexagon along with six cavities by using a 5 mm steel plate, were the same. The vertical continuous stiffening ribs along with a cross-section of 25 mm × 3 mm were welded to the inside face of an external steel tube and to the both faces of the partition steel plates. The studs along with a diameter of 4 mm, a length of 30 mm and a spacing 60 mm×60 mm were welded to the same place as the vertical continuous stiffening ribs. For the column specimens CFT1-P, CFT3-P, CFT1-H and CFT3-H, the longitudinal reinforcement is arranged into cavities by using a welded spacer bar to improve the shrinkage of mass concrete and the heat of hydration issues as well as to constrain the inner concrete. The main parameters and the running parameters of the six specimens have been figured out, as these have been shown in Table 1. The construction details have also been shown in Figure 2, whereas the construction photos have been shown in Figure 3.

**Table 1:** Main parameters of the specimens.

Group	Name	Shape	Quantity of Cavities	Cross-Sectional Area	Concrete Strength		Equivalent Steel Strength	Steel Plate Ratio		Steel-Bars Ratio
				*A* (m^2^)	*f*_cu,m_ (Mpa)	*f*_c,m_ (Mpa)	$\bar{f_{y}}$ (Mpa)	ρ_1_ (%)	ρ_2_ (%)	ρ_3_ (%)
P	CFT1-P	Irregular pentagon	1	0.354	51.1	38.8	378.5	7.81	1.68	0.29
	CFT2-P		4				385.8	7.81	3.60	0
	CFT3-P		4				385.7	7.81	3.60	0.29
H	CFT1-H	Irregular hexagon	6	0.313	30.7	23.3	300.5	3.42	2.60	0.82
	CFT2-H		6		42.0	31.9	295.0	3.42	2.60	0
	CFT3-H		6		42.0	31.9	300.5	3.42	2.60	0.82

Note: *f*_cu,m_ is the tested average concrete cubic strength (150 mm × 150 mm × 150 mm); *f*_c,m_ = 0.76*f*_cu,m_ is the average concrete axial compressive strength [25]; $\bar{f_{y}}=\frac{\sum f_{yi}A_{si}}{\sum A_{si}}$ is the equivalent steel strength; ρ_1_ is external steel tube steel plate ratio; ρ_2_ is inner partition steel plate ratio.

#### 3.1.2. Material Properties

The concrete strength has been shown in Table 1. The tested yield strength, the ultimate strength, the tensile elongation, the elasticity modulus of reinforcement and the steel plates have been shown in Table 2.

**Table 2:** Mechanical properties of reinforcement and steel plates.

Group	Type	Location	*f*_y_ (MPa)	*f*_u_ (MPa)	ρ (%)	*E*_s_ (MPa)
P	6 mm steel plate	Vertical and horizontal stiffening ribs, lattice partition steel plate	416	528	27.5	2.10 × 10^5^
	10 mm steel plate	Solid partition steel plate	409	498	27.6	2.12 × 10^5^
	12 mm steel plate	External steel tube	373	525	27.4	2.06 × 10^5^
	ø6 reinforcement	Longitudinal reinforcement	382	582	31.3	2.07 × 10^5^
	ø10 reinforcement	Longitudinal reinforcement	310	473	36.7	2.05 × 10^5^
H	5 mm steel plate	Steel tube	296	428	28.9	2.06 × 10^5^
	ø8 reinforcement	Longitudinal reinforcement	334	445	24.5	2.05 × 10^5^
	ø10 reinforcement	Longitudinal reinforcement	363	446	26.3	2.07 × 10^5^
	ø12 reinforcement	Longitudinal reinforcement	326	423	27.1	2.04 × 10^5^

Note: *f*_y_ is the tested yield strength; *f*_u_ is tested ultimate strength; ρ is the tested tensile elongation; *E*_s_ is the tested elastic modulus of steel.

#### 3.1.3. Experimental Set-Up

A 40,000 kN universal testing machine was used to conduct the axial compressive tests. The axial load is applied at the centroid of the cross-section. The coordinate of the centroid is calculated by formulas $x=\sum \sigma_{i}A_{i}x_{i}/\sum \sigma_{i}A_{i}$ and $y=\sum \sigma_{i}A_{i}y_{i}/\sum \sigma_{i}A_{i}$, where $\sigma_{i}$ is the strength of concrete part or steel part, and $A_{i}$ is the area of concrete part or steel part. On the upper and the lower end of the loading device, the spherical hinges have been arranged. During the testing, a cyclically uniaxial load was applied to the specimens to study the residual deformation each time the unloading was finished. To prevent the overturn of the specimens, the device was unloaded to 2000 kN. During the initial stage (*i.e.*, the elastic stage), the specimens were loaded at the intervals of one-sixth an estimated ultimate load. After evident yield appeared on the load-displacement curves, the loading process principle turned out to be controlled by displacement.

**Figure 4:**
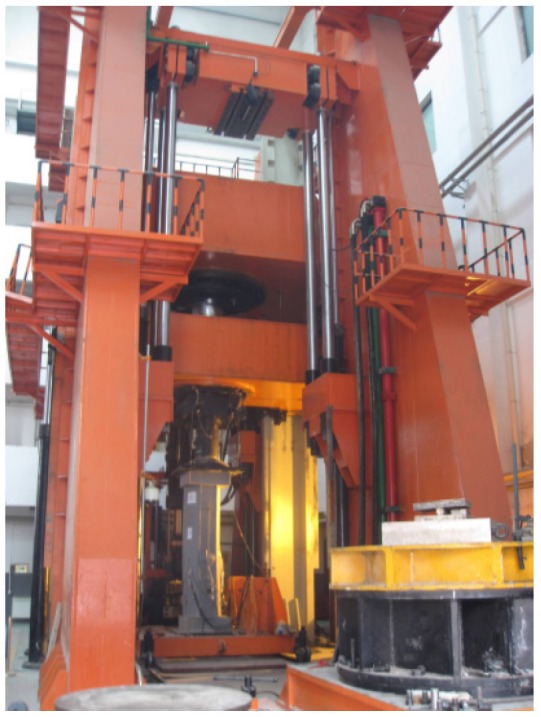
Test scene.

**Figure 5:**
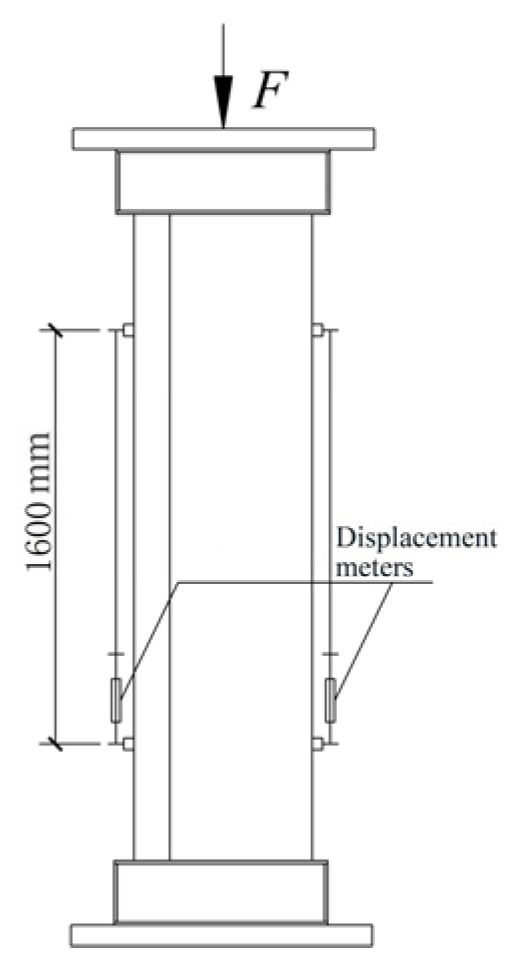
Displacement meters arrangement.

**Figure 6:**
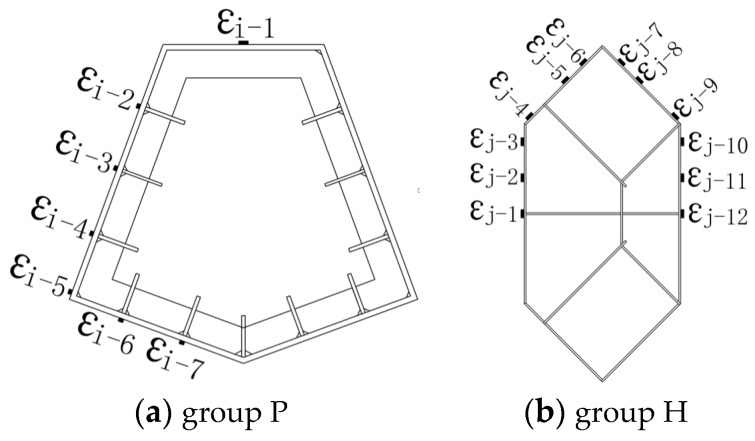
The arrangement of strain gauges.

The two displacement meters, which were used to measure the vertical displacement, were arranged in the central part of the specimens, where the deformation was uniform. The gauge length of the displacement meters is 1600 mm. The strain gauges to measure longitudinal deformation were also placed on the exterior of steel plates of central steel tubes in the vertical direction. The real-time values of the load, the displacement and the strain were gathered by using a data gathering system; the buckling of external steel tube and crack of welding seams were recorded manually. The test scene photo has been shown in Figure 4. The arrangement of displacement meters has been shown in Figure 5; the distribution of the strain gauges has also been shown in Figure 6.

#### 3.1.4. Test Phenomenon

**Figure 7:**
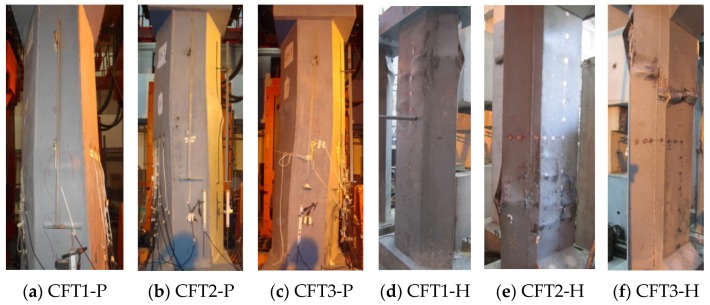
Failure patterns of specimens.

All the specimens went through a similar failure process, *i.e.*, wrinkling of oil painted skin, buckling of steel plates, cracking of welding seams, breaking of concrete, *etc.* The final failure patterns have been shown in Figure 7.

There are few differences between the two groups of specimens. The wrinkling of oil painted skin in group P specimens is horizontal cracks, while that in group H specimens is 45-degree staggered cracks. It shows that the vertical strain develops faster than the hoop strain in group P specimens, while the vertical strain develops close to the hoop strain in group H specimens. The buckling regions of group P specimens are few and concentrate in only two to three regions, but each buckling region is large; by contrast, the buckling regions of group H specimens are numerous and scattered, but each local buckling region is small and protruding. There are great differences between the two groups of specimens. The reason is that the steel ratio of group P specimens is higher than that of group H specimens by 58.63% to 85.05%. The cross sectional moment of inertia of group P specimens in the two main directions is close to each other, whereas it is not close to each other in the case of group H specimens. Owing to an arrangement of strong vertical and horizontal stiffening ribs, the stability of external steel tubes of group P specimens is better and the confinement effect to infill concrete is stronger.

#### 3.1.5. Load *F*-Average Strain ε Curves

**Figure 8:**
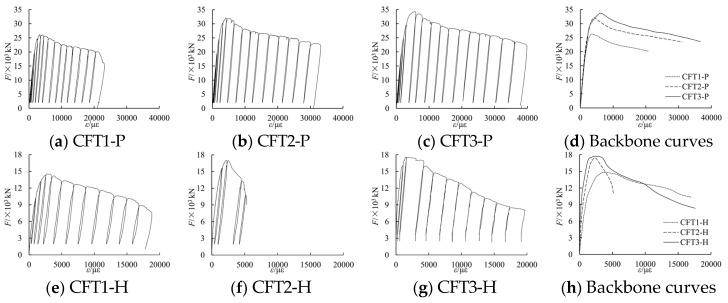
Tested *F*-ε curves and backbone curves of specimens.

Under a cyclically uniaxial load, the tested load *F*-average strain ε curves and the related backbone curves of the six columns have been shown in Figure 8. In Figure 8, *F* is the applied axial load during the testing process, whereas ε is the average strain that is switched from the related displacement in the 1600 mm gauge length of the middle of columns. The tested characteristic points have been shown in Table 3. In Table 3, *F*_ut_ is the tested peak load, whereas ε_ut_ is the tested strain related to *F*_ut_; *F*_u0_ is the aggregate value bearing capacity *s* of concrete and the steel part.

**Table 3:** Peak results of specimens.

Columns	CFT1-P	CFT2-P	CFT3-P	CFT1-H	CFT2-H	CFT3-H
*F*_ut_/kN	26233	32119	33496	14800	17400	17557
ε_ut_/με	3316	4049	5650	3144	2495	2800
*F*_u0_	24370	26268	26612	12636	14343	15138

#### 3.1.6. Backbone Curves of Load *F*-Measured Strain ε*_i_*

**Figure 9:**
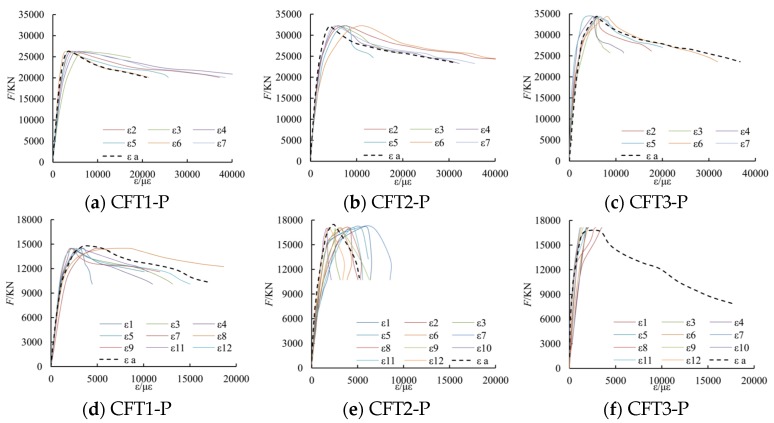
Backbone curves of *F*-ε*_i_*.

Parts of the tested backbone curves of load *F*-measured strain ε*_i_* curves are shown in Figure 9. In the figure, ε_a_ is the tested average strain switched from the related displacement.

From the figure, it can be known that the change law of the measured stain by strain gauges is similar to the average stain switched from the related displacement. Moreover, when the longitudinal deformation of the columns reaches a larger value, local buckling of the steel plate may occur, so that the strain measured by strain gauges may not be accurate any more. Thus, in this paper, in order to simply calculations and reduce errors, the average strain switched from the related displacement is applied to study the relationship of the concrete confined by special-shaped steel tube coupled with multiple cavities.

### 3.2. Determination of the Effective Confinement Coefficient k_e_

The confinement action of core concrete that is confined by a special-shaped CFT coupled with multiple cavities is different from one that is confined by normal reinforcement. The external steel tube has strong confinement action at the corners and the locations that were provided with partition steel plates, longitudinal stiffening ribs or transversal stiffening ribs. The confinement action is relatively weak at the central part of the external steel tube plate that may exist either between the two adjacent longitudinal stiffening ribs or between the adjacent longitudinal stiffening rib and the partition steel plate. Therefore, the cross-sectional boundary in transversal direction between the active confined region and the inactive confined region is assumed to be a parabola, the same as occurs in lateral cross-section due to similar confinement features between the two adjacent transverse stiffening ribs.

The cavity construction and the arrangement of stiffening ribs in two group columns differ from each other. If the arranged stiffening ribs are weak, the confinement action of the steel tube to the core concrete is relatively weak. In that case, the concrete near to the stiffening ribs cannot be divided into the active confined region. Keeping this in view, specific criteria has been proposed to evaluate whether the stiffening ribs are able to offer sufficient confinement ability or not. Firstly, the division of active and inactive confined regions is based on the straight sides of the external steel tube. In case the boundary parabola intersects all the longitudinal stiffening ribs whose width to thickness ratios are relatively small, sufficient confinement ability can preliminary be verified. Secondly, if the boundary parabola only intersects parts of the longitudinal stiffening ribs, a parameter *S* describing the contribution of stiffening ribs of the cavity side on the whole cavity is additionally used to verify the confinement ability. Similarly, the confinement ability of transversal stiffening ribs has been assured by *S*. The expression of parameter *S* can be written as follows:

$S=\frac{A_{j}}{\frac{b_{j}}{c_{j}}A_{aj}}\times 100\%$, where *A_j_* is the cross-sectional area of stiffening ribs on one cavity side, *b_j_* is the length of cavity side, *c_j_* is the perimeter of the cavity, and *A_aj_* is the cross-section area of the cavity.

**Figure 10:**
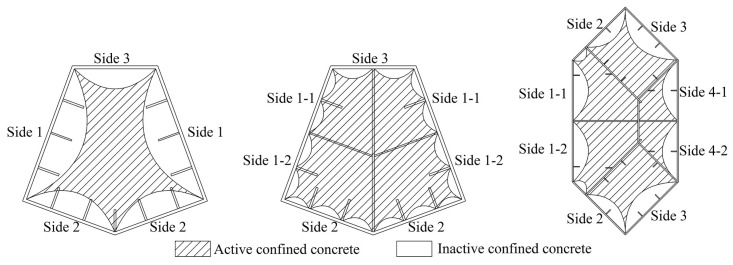
The active confined concrete and inactive confined concrete.

The issue of confinement ability of stiffening ribs is very complex and needs extensive research. Additionally, the samples of experimental column are limited, so the validity of stiffening ribs can be determined on a qualitative basis only. The estimated results have been shown in Table 4. The division of active and inactive confined concrete has been shown in Figure 10.

**Table 4:** The validity determination of stiffening ribs.

Columns	Evaluating Parameters	Side Number	CFT1-P	CFT2-P	CFT3-P	Side Number	CFT1-H	CFT2-H	CFT3-H
Longitudinal stiffening ribs	*b*/*t*	-	15	15	15	-	8.3	8.3	8.3
	*S*/%	1-1	1.958	3.192	3.192	1-1 (1-2)	0.850	0.850	0.850
		1-2		2.530	2.530	2	0.787	0.787	0.787
		2	1.948	3.809	3.809	3	0.526	0.526	0.526
		3	-	-	-	4-1 (4-2)	0.542	0.542	0.542
	Validity	-	Inactive	Active	Active	-	Inactive	Inactive	Inactive
Transverse stiffening ribs	*b*/*t*	-	15	15	15	-	-	-	-
	*S*/%	1-1	0.435	3.629	3.629	-	-	-	-
		1-2		2.128	2.128				
		2	0.649	1.686	1.686				
		3	0.743	1.270	1.270				
	Validity	-	Inactive	Active	Active	-	-	-	-

**Figure 11:**
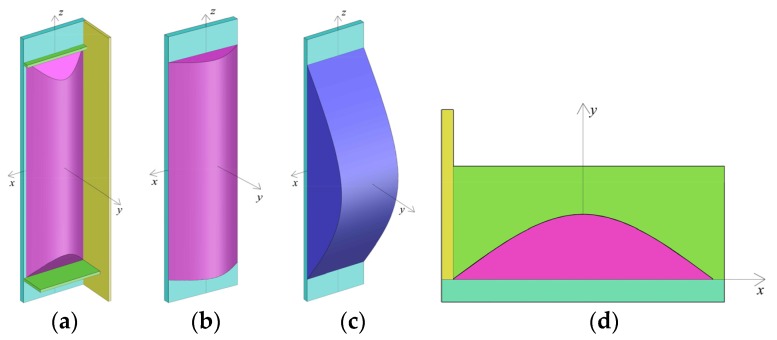
Schematic diagram of 3D boundary of inactive and active cofined concrete. (**a**) Isolated body; (**b**) Transverse boundary; (**c**) Lateral boundary; (**d**) Plane graph.

To determine the extent to which concrete is confined actively, a local coordinate system o-*xyz* has been defined, as it has been shown in Figure 11. The *x* axis is parallel to the straight side; the *y* axis is perpendicular to the straight side; and the *z* axis is in the longitudinal direction of the column. The distance either between the adjacent effective longitudinal stiffening ribs or between the adjacent effective longitudinal stiffening rib and the partition steel plate is *b*. The distance between the adjacent effective transversal stiffening ribs is regarded as *H*. If the points of intersection between the parabola and steel plate side are (−*b*/2, 0) and (*b*/2, 0) and the included angle between the tangent line of the parabola and steel plate side at the points of intersection is θ, the transversal boundary between the active confined concrete and the inactive confined region can be expressed by using the relation: $y=f(x)=-\frac{\tan\theta }{b}x^{2}+\frac{b}{4}\tan\theta$. Analogously, the lateral boundary can be expressed by using the relation: $y=g(z)=-\frac{\tan\theta }{h}z^{2}+\frac{h}{4}\tan\theta$.

The maximum value of *f*(*x*) and *g*(*z*) may not be equal to each other. When *H* is greater than that of *b*, *g*(*z*)_max_ is greater than that of *f*(*x*)_max_, and the transversal boundary differs from the lateral boundary. It shows that the confinement action in transversal cross-section is stronger than that of the lateral cross-section. The transversal confinement action is sufficient; therefore, the transverse boundary can be regarded as a primary boundary.

It is logical to know that when $z=\pm \frac{\sqrt{(h-b)h}}{2}$, then *g*(*z*) = *f*(*x*)_max_ = $\frac{h}{4}\tan\theta$. Therefore, the 3D boundary of active and inactive confined concrete can be expressed as follows:

When $-\frac{h}{2}<z<-\frac{\sqrt{(h-b)h}}{2}$ or $\frac{\sqrt{(h-b)h}}{2}<z<\frac{h}{2}$, the included angle between the tangent line of the parabola *f*(*x*) and the axis at the intersection points changes along with the *z* value. The *f*(*x*)_max_ can be determined by using the relation *g*(*z*). The parabola *f*(*x*) intersects xz plane at points (−*b*/2, 0, *z*) and (*b*/2, 0, *z*); therefore, the transversal boundary can be modified to be included as: $y=f(x)=-\frac{4}{b^{2}}g(z)x^{2}+g(z)$.

When $-\frac{\sqrt{(h-b)h}}{2}<z<\frac{\sqrt{(h-b)h}}{2}$, the transversal boundary cannot be modified and the expression is considered as: $y=f(x)=-\frac{\tan\theta }{b}x^{2}+\frac{b}{4}\tan\theta$.

As a result, the final 3D boundary can be written as:

(6) $$y=t(x,z)=\{\begin{array}{c} -\frac{4}{b^{2}}g(z)x^{2}+g(z),\;-\frac{h}{2}<z<-\frac{\sqrt{(h-b)h}}{2}\\ -\frac{\tan\theta }{b}x^{2}+\frac{b}{4}\tan\theta ,\;-\frac{\sqrt{(h-b)h}}{2}<z<\frac{\sqrt{(h-b)h}}{2}\\ -\frac{4}{b^{2}}g(z)x^{2}+g(z),\;\frac{\sqrt{(h-b)h}}{2}<z<\frac{h}{2} \end{array}$$

To simplify the estimation, a constant value of θ = 45° is applied [26]. The volume of the inactive confined concrete can be calculated by using Equation (7). In the estimation process, the distance between the two adjacent active transversal stiffening ribs can be regarded as an effective length *H*. If there are no active transversal stiffening ribs, the whole length of column can be regarded as an effective length.

(7) $$V_{\text{in}}=\iint t(x,z)dxdz,-\frac{h}{2}<z<\frac{h}{2},-\frac{b}{2}<x<\frac{b}{2}=\frac{b^{2}\tan\theta \sqrt{h(h-b)}}{6}+\frac{b\tan\theta \left[2h^{2}-(2h+b)\sqrt{h(h-b)}\right]}{18}$$

The coefficient of active confinement can be calculated by using Equation (8). In Equation (8), *V*_c_ presents the whole volume of the concrete in the columns within the effective length *H*.

(8) $$k_{e}=\frac{V_{c}-\sum V_{\text{in}}}{V_{c}}$$

when *H* is smaller than that of *b*, *g*(*z*)_max_ is smaller than that of *f*(*x*)_max_. The confinement action in transversal cross-section is weaker than that of the lateral cross-section. In addition, the estimation method of *k*_e_ is similar to that when *H* is greater than *b*. Moreover, in general design of special-shaped CFT coupled with multiple cavities, *h* < *b* is unusual.

### 3.3. Determination of Hooping Stress f_sr_ and Longitudinal Stress f_a_

For a general design, the issue of local buckling for steel plates is usually considered, and the steel tube is often found in a relatively balanced state. Hence, united parameter is applied to describe the issue of local buckling. By neglecting the radial stress, the steel plate of tube is assumed to be in a plane stress state and it accords with von Mises yield criterion. Ge [27] shows that the parameter of width to thickness *R* is a major factor that influences the damage of in-filled concrete. When *R* > 0.85, the local buckling may appear before the applied load reaches the ultimate bearing capacity; when *R* ≤ 0.85, the issue of local buckling may be neglected, but the longitudinal stress of steel tube can not reach the yielding stress owing to the opposite sign of stress fields.

The width to thickness parameter of each side in the isolated body has been shown in Equation (9), where *t* is the thickness of steel plate and, *ν* is the Poisson ratio.

(9) $$R_{i}=\frac{b_{i}}{t}\sqrt{\frac{12(1-\nu^{2})}{4\pi^{2}}}\sqrt{\frac{f_{y}}{E}}$$

The equivalent width to thickness parameter of the special-shaped steel tube with multiple cavities has been shown in Equation (10).

(10) $$\bar{R}=\frac{\sum R_{i}b_{i}}{\sum b_{i}}$$

When *R* > 0.85, the yielding strength of steel tube plate, due to local buckling, can be assessed by using Equation (11).

(11) $$\frac{f_{a}}{f_{y}}=\frac{1.2}{R_{i}}-\frac{0.3}{R_{i}^{2}}\leq 1.0$$

During estimation, *ν* = 0.283 [13], even though the stiffening ribs in columns CFT1-H–CFT3-H are inactive in the division of active and inactive region of confined concrete, they are active in considering local buckling of the steel tube plate. The estimated results have been shown in Table 5, and the issue of local buckling for all the columns in this article does not need to be considered.

By neglecting the issue of local buckling, Sakino *et al.* [28] have suggested that the hooping stress *f*_sr_ and the longitudinal stress *f*_a_ can be assumed as 0.19*f*_y_ and −0.89*f*_y_ respectively, whereas Architectural Institute of Japan (AIJ) [29] standard suggested that the hooping stress *f*_sr_ and the longitudinal stress *f*_a_ can be assumed as 0.21*f*_y_ and −0.89*f*_y,_ respectively. By considering the non-uniformity of the special-shaped CFT coupled with multiple cavities in this paper, the smaller values of *f*_sr_ = 0.19*f*_y_ was applied in the estimation process.

**Figure 12:**
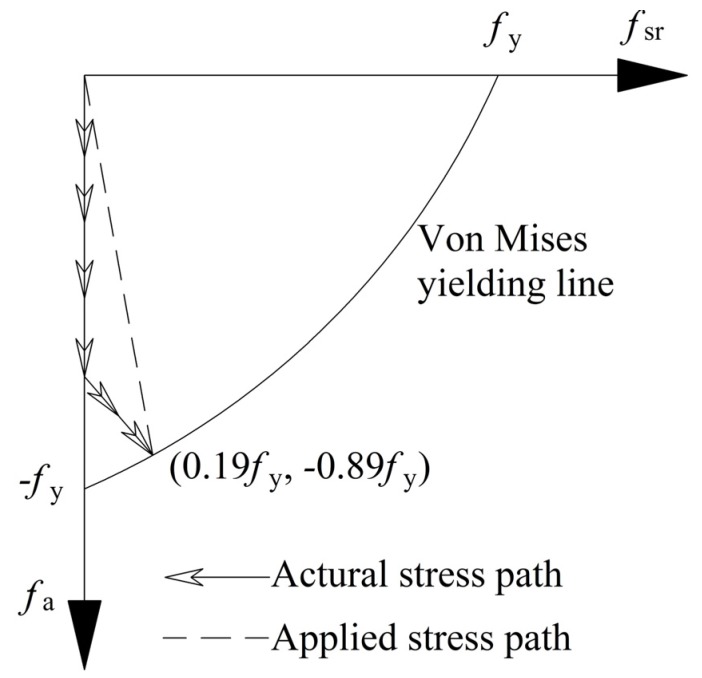
Curve of hooping and longitudinal stresses of steel tube.

To simplify the estimation, the stress path of steel tube plate was simplified into a straight line, as has been shown in Figure 12.

**Table 5:** Estimated results of related parameters of confined concrete.

Columns	CFT1-P	CFT2-P	CFT3-P	CFT1-H	CFT2-H	CFT3-H
*k*_e_	0.461 (0.498)	0.856	0.856 (0.498)	0.699	0.702	0.699
$\bar{R}$	0.346	0.255	0.255	0.465	0.465	0.465
*f*_l_	3.563 (0.648)	4.692	4.692 (0.648)	2.473	2.473	2.473
*f*_l_	1.641 (0.323)	4.017	4.015 (0.323)	1.729	1.735	1.729
ξ_1_	0.8309	0.8476	0.8503	0.4638	0.3361	0.3391
ξ_2_	0.0255	0	0.0316	0.1290	0	0.0943
ξ_3_	0.1991	0.8992	0.9021	0.7075	0.5126	0.5172
ξ	1.0555	1.7468	1.7840	1.3003	0.8487	0.9506
η	3.800	2.796	2.855	2.353	1.528	1.720
*f*_c0_	38.84	38.84	38.84	23.32	31.90	31.90
ε_c0_	1772	1772	1772	1531	1671	1671
*E*_c_	32831	32831	32831	26269	30753	30753
*f*_cc_	49.12 (51.01)	61.41	61.40 (62.87)	33.55	42.57	42.53
ε_cc_	3565 (3881)	4654	4709 (4901)	3111	2523	2629
*r*	1.725 (1.718)	1.673	1.659 (1.705)	1.696	2.213	2.109
*F*_ut_/kN	27312	33343	33760	15350	17470	18343
*F*_ut_/*F*_ut_	1.041	1.038	1.008	1.037	1.004	1.045
ε_cc_/ε_ut_	1.075	1.149	0.834	0.990	1.011	0.939

Note: The values as shown in brackets are meant for multi-confined concrete by steel tube and effective reinforcement.

### 3.4. Determination of Equivalent Tensile Force F_b_ of Partition Steel Plate at Peak Point

The stress status of a solid partition steel plate is similar to that of steel tube. The radial stress can be neglected, as it is relatively small in comparison with longitudinal and hooping stresses. It is assumed that the partition steel plate can be in a plane stress status and accords with von Mises yield criterion. Both sides of the steel plate are constrained by concrete. Therefore, the issue of local buckling can be avoided. The estimation method of longitudinal and hooping stresses can be used to refer to the steel tube plate when *R* < 0.85. In addition, the stress path can be simplified into a straight line.

The stress status of latticed partition steel plate is very complex. If the radical stress is neglected, the solid part of lattice partition steel plate can be assumed to be in a plane stress status, whereas the latticed part can be assumed to be in a one-dimensional stress status. When the applied load reaches to the ultimate bearing capacity, the equivalent tensile force of latticed partition steel plate can be estimated by Equations (12)–(14), where *A_b1_* is the area of solid part, *A_b2_* is the area of latticed part, and ε*_b2_* is the strain of latticed part that is corresponded to peak load.

(12) $$F_{b}=\min\left(F_{\text{b}1},F_{\text{b}2}\right)$$

(13) $$F_{\text{b}1}=A_{\text{b}1}\times 0.19f_{y}$$

(14) $$F_{\text{b}2}=A_{\text{b}2}E_{\text{b}2}\varepsilon_{\text{b}2}<A_{\text{b}2}f_{y}$$

The deformation compatibility condition can be met by assuming that there is no slippage between the lattice partition steel plate and the concrete. The equivalent tensile strain of the lattice partition steel plate can be taken as $\varepsilon_{\text{b}2}=\mu_{c}\varepsilon_{c}$.

Han *et al.* [14] proposed the expression of the Poisson ratio of a core concrete at peak that was point based on axial compressive experimental research on plain concrete and CFT stud columns, as has been shown in Equation (15).

(15) $$\mu_{c}=0.173+\left[0.7306{\left(\frac{\sigma_{c}}{f_{\text{cc}}}-0.4\right)}^{1.5}\left(\frac{f_{\text{ck}}}{24}\right)\right]$$

Estimation results show that the longitudinal solid plate part of the lattice partition steel plate is not able to provide enough transversal tensile stress. Therefore, the equivalent tensile force of the latticed partition steel plate can be assessed by *F*_b_ = *F*_b1_.

### 3.5. Determination of Equivalent Lateral Confining Stress of Concrete f*_l_*

The confinement action of a special-shaped steel tube coupled with multiple cavities is not uniform at the corners and at the center of the straight sides. To make it convenient to estimate, the confining stress of a special-shaped steel tube coupled with multiple cavities is equivalent to uniformly distribute and an active confinement coefficient *k*_e_ is multiplied to reflect the influence of non-uniformity. During the calculation, the external steel tube plate of each cavity is taken as an isolated body. An equivalent average confining stress of all the external steel tube in each cavity can be gained by using the equilibrium condition.

**Figure 13:**
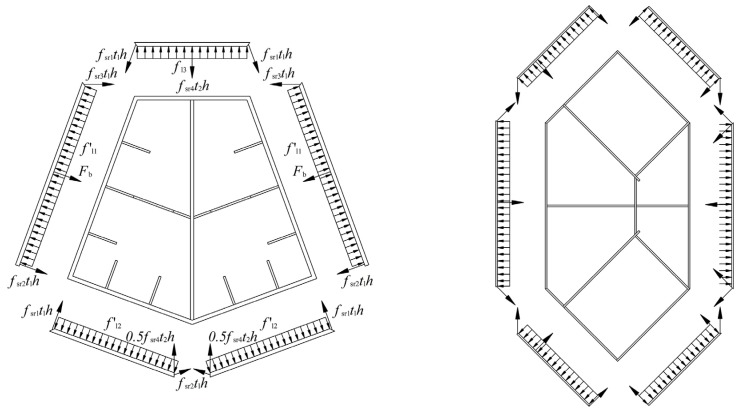
The diagrams of the isolated bodies of the specimens.

The diagrams of isolated bodies of the two group columns are shown in Figure 13. Equations (16)–(20) of group P are given, for instance.

(16) $$f_{\text{l}1}^{\prime }b_{1}h=f_{\text{sr}2}t_{1}h+f_{\text{sr}3}t_{1}h\sin70{}^{\circ}+F_{b}$$

(17) $$f_{\text{l}2}^{\prime }b_{2}h=f_{\text{sr}1}t_{1}h+0.5f_{\text{sr}4}t_{2}h\sin70{}^{\circ}$$

(18) $$f_{\text{l}3}^{\prime }b_{3}h=2f_{\text{sr}1}t_{1}h\sin70{}^{\circ}+f_{\text{sr}4}t_{2}h$$

(19) $$f_{l}^{\prime }=\frac{2f_{l1}^{\prime }b_{1}+2f_{l2}^{\prime }b_{2}+f_{l3}^{\prime }b_{3}}{2b_{1}+2b_{2}+b_{3}}$$

(20) $$f_{l}=k_{e}f_{l}^{\prime }$$

For the columns CFT1-P, CFT3-P, CFT1-H and CFT3-H, the reinforcement is arranged in the cavities. The concrete rounded by reinforcement is confined by both reinforcements and special-shaped steel tube coupled with multiple cavities. Han [14] shows that the stress of concrete near center is higher than that near straight sides for normal steel tube confined concrete. Thus, the confinement action of the concrete rounded by reinforcement in this paper is stronger than the average confinement action. To reflect this feature, a sum of the steel tube confining stress and the reinforcement confining stress, which are estimated by Mander’s methods, are applied as equivalent confining stress. The multiple confinement action is limited for the column CFT1-H and CFT3-H owing to the reason that no hooping reinforcement has been arranged. The reinforcement confining stress can be assessed by Equation (21) [22], where ρ_s_ is ratio of the volume of transversal confining steel to the volume of confined concrete core, *f*_yh_ is the yield strength of the transversal reinforcement, *s* is the clear vertical spacing between spiral or hoop bars, *d*_s_ is the diameter of spiral between the bar centers, and ρ_cc_ is the ratio of area of longitudinal reinforcement to area of core of section.

(21) $$f_{l}=\frac{1}{2}\rho_{s}f_{\text{yh}}\frac{{\left(1-\frac{s^{\prime }}{2d_{s}}\right)}^{2}}{\left(1-\rho_{\text{cc}}\right)}$$

### 3.6. Determination of Modified Factor of Strain at Maximum Concrete Stress η

The modified factor of strain at maximum concrete stress η is a constant value in Mander’s confined concrete model. In fact, the value of η varies along with the confinement action. In a special-shaped CFT coupled with multiple cavities, the value of η is related to cross-section shape, stiffening ribs, cavity construction, concrete strength and steel strength. After comprehensive analysis, it can be observed that the influence of cross-sectional shape, stiffening ribs and cavity construction have been reflected by the parameter *k*_e_. Thus, the remaining factors can be reflected by material confinement coefficient ξ. The parameter ξ is divided into three parts as follows: (1) the first part ξ_1_ is the coefficient of material confinement for the external steel tube; (2) the second part ξ_2_ is the coefficient of material confinement for reinforcement; and (3) the third part ξ_3_ is the coefficient of material confinement for partition steel plate and stiffening ribs. The parameter ξ_3_ can be determined by using the material strength, the area, and the share times of cavities. For instance, if a part or whole partition steel plate is shared by two cavities, during the estimation of ξ_3_, the area of the part or whole partition steel plate can be multiplied by two; if the longitudinal stiffening ribs are verified as effective, the area would be multiplied by two; except for the above situation, the area would be multiplied by one during the estimation of ξ. The equation of ξ are as follows:

(22) $$\xi =\xi_{1}+\xi_{2}+\xi_{3}$$

(23) $$\xi_{1}=\frac{A_{e}f_{ye}}{A_{c}f_{c,m}}$$

(24) $$\xi_{2}=\frac{A_{r}f_{yr}}{A_{c}f_{c,m}}$$

(25) $$\xi_{3}=\frac{\sum n_{i}A_{i}f_{yi}}{A_{c}f_{c,m}}$$

**Figure 14:**
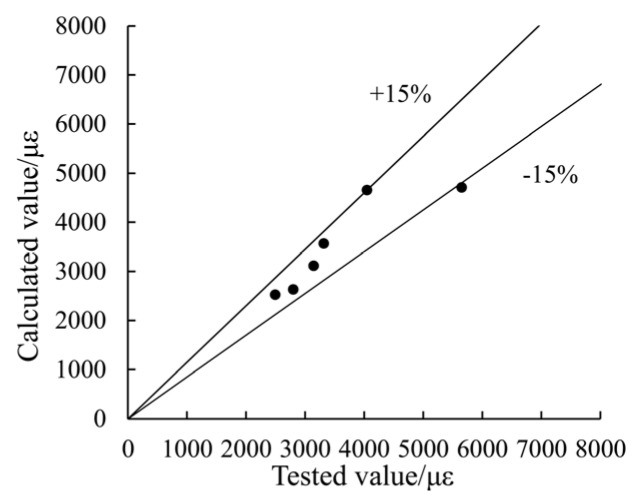
A comparison between the estimated values and the tested values of strain at maximum concrete stress.

After regression analysis of experimental data containing the six columns, the parameter η is derived as shown in Equation (26). The estimated strain at maximum concrete stress matches well to the test results, as has been shown in Figure 14.

(26) $$\eta =\left(15.596k_{e}^{2}-25.590k_{e}+12.077\right)\xi$$

## 4. Comparison between the Predicted and the Experimental Axial Load-Strain Curves

### 4.1. Stress-Strain Relationship of Steel

**Figure 15:**
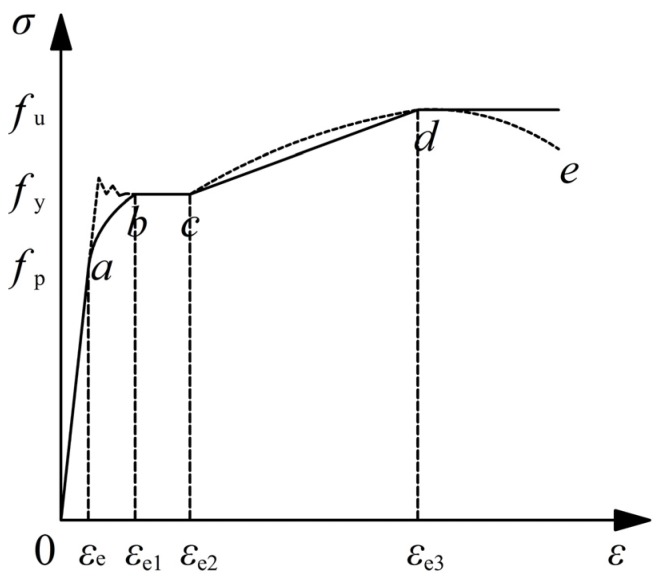
Simplified stress-strain relationship for steel tube and reinforcement.

For the cases of common low-carbon soft steel and structural low alloy steel in building engineering, the stress-strain curves can be divided into five stages including the elastic stage, the elastic-plastic stage, the plastic stage, the strengthening stage, and the secondary plastic flow stage [13], as have been shown in Figure 15. In the figure, the imaginary line is the actual stress-strain curve and the solid line is the simplified curve. The equation of the simplified curve has been shown as follows:

(27) $$\sigma_{s}=\{\begin{array}{cc} E_{s}\varepsilon_{s}, & \varepsilon_{s}\leq \varepsilon_{e}\\ -A\varepsilon_{s}^{2}+B\varepsilon_{s}+C, & \varepsilon_{e}\leq \varepsilon_{s}\leq \varepsilon_{\text{e}1}\\ f_{y}, & \varepsilon_{\text{e}1}\leq \varepsilon_{s}\leq \varepsilon_{\text{e}2}\\ f_{y}\left[1+0.6\frac{\varepsilon_{s}-\varepsilon_{\text{e}2}}{\varepsilon_{\text{e}3}-\varepsilon_{\text{e}2}}\right], & \varepsilon_{\text{e}2}\leq \varepsilon_{s}\leq \varepsilon_{\text{e}3}\\ 1.6f_{y}, & \varepsilon_{s}>\varepsilon_{\text{e}3} \end{array}$$

In Equation (27), ε_e_ = 0.8*f*_y_/*E*_s_, ε_e1_ = 1.5*ε*_e_, ε_e2_ = 10*ε*_e1_, ε_e2_ = 100*ε*_e1_, *A* = 0.2*f*_y_/(ε_e1_ − ε_e_)^2^, and *B* = 2*A*ε_e1_, *C* = 0.8*f*_y_ + *A*ε_e2_ − *B*ε_e_.

### 4.2. Parameters and Stress-Strain Curves of Concrete

**Figure 16:**
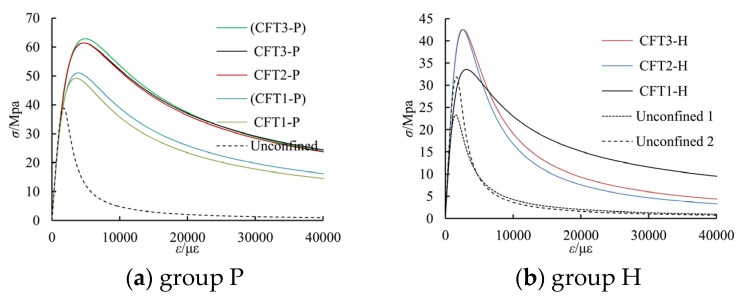
The estimated stress-strain relationship for confined concrete.

In accordance with the estimation methods as described earlier, the related parameters of the concrete for the special-shaped CFT coupled with multiple cavities, as studied in this article, are shown in Table 5. The stress-strain curves, as estimated by Equation (1), are shown in Figure 16; in the figure, the stress-strain curves of the unconfined concrete are given based on Chinese National Standard GB50010-2010 [25].

### 4.3. Load-Longitudinal Strain Relationship Curves

**Figure 17:**
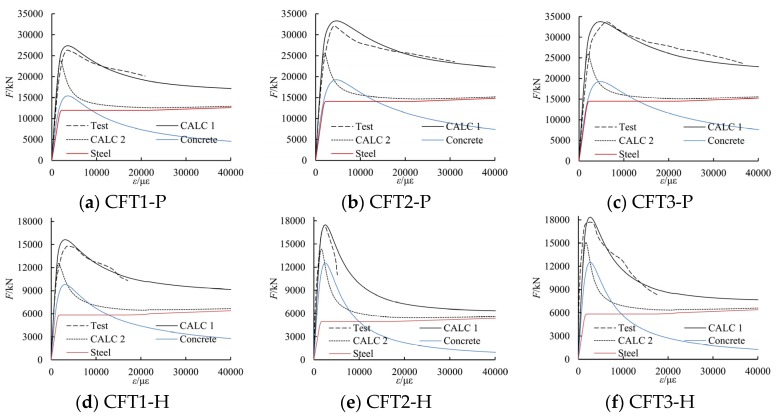
The estimated curves of load-longitudinal strain relationship.

The load-strain relationship curves of the six special-shaped CFT columns coupled with multiple cavities are calibrated by using a fiber-based model and by using the tested data. During the estimation process, it is assumed that there is no slip between the steel tube and the core concrete, and only the conditions of longitudinal equilibrium and deformation compatibility are considered. The estimated load-strain relationship curves have been shown in Figure 17. In the figure, CALC 1 represents the estimation results by the method considering concrete confinement effect in this article, while CALC 2 represents the estimation results by the method neglecting the concrete confinement effect. As shown in Figure 17, the estimated curves by the model in this article are found to be in good agreement with the test results. This agreement leads to the conclusion that the proposed model of concrete can be used to evaluate the non-linear behavior of special-shaped CFT columns with multiple cavities subjected to axial load.

## 5. Conclusions

In order to estimate the confinement effect of the special shaped CFT columns coupled with multiple cavities subjected to axial compressive load in real high-rise buildings, this article develops a model of uniaxial stress-stain relationship of the confined concrete. It is pertinent to mention that all factors, *i.e.*, cross-sectional shape, cavity construction, steel ratio of outer steel tube, steel ratio of inner cavity partition steel plate, steel ribs, and steel bars, are all considered in this model. The estimated complete curves of load-strain matches the test results well

Hence, it can be concluded that the proposed constitutive relationship for the confined concrete can be used well to predict the non-linear compressive behavior of special-shaped CFT columns with multiple cavities. Next, the cavity construction and reinforcement in cavities make it possible to form a combined constraint to in-filled concrete, so the confinement effect cannot be neglected.

Although this article uncovers important findings regarding the constitutive relationship of confined concrete in special-shaped CFT columns with multiple cavities, it is still required to explore the following: (1) the parameterized effectiveness estimation method of stiffening ribs in the division of active confined regions and inactive confined regions; (2) the reasonable design methods of special-shaped CFT with multiple cavities, *etc.*
